# Association of trabecular bone score corrected for tissue thickness with glucose metabolism in acromegaly

**DOI:** 10.3389/fendo.2024.1448566

**Published:** 2024-12-12

**Authors:** Martin Kužma, Peter Vaňuga, Dušan Pávai, Zdenko Killinger, Didier Hans, Neil Binkley, Juraj Payer, Peter Jackuliak

**Affiliations:** ^1^ 5th Department of Internal Medicine, Comenius University Faculty of Medicine, University Hospital Bratislava, Bratislava, Slovakia; ^2^ Department of Endocrinology, National Institute of Endocrinology and Diabetology, Lubochna, Slovakia; ^3^ Center of Bone Diseases, Bone and Joint Department, Lausanne University Hospital, Lausanne, Switzerland; ^4^ Department of Medicine, University of Wisconsin, Madison, WI, United States

**Keywords:** acromegaly, glucose metabolism, trabecular bone score, tissue thickness, bone mineral density

## Abstract

**Introduction:**

Acromegaly is associated with increased vertebral fracture (VF) risk regardless of bone mineral density (BMD). However, the vertebral trabecular compartment is still low; a possible contributor to this may be impaired glucose metabolism (GM) which frequently complicates acromegaly. Additionally, soft tissue thickness may confound bone imaging in acromegaly patients.

**Objective:**

This study aims to assess the association of GM with BMD, trabecular bone score adjusted for BMI (TBS_BMI_), and trabecular bone score adjusted for tissue thickness (TBS_TT_) among acromegaly subjects.

**Patients and methods:**

A cross-sectional study was performed among 70 consecutive acromegaly patients (24 male/46 female, aged 55.1 years) divided in two subgroups: abnormal GM (*n* = 35) and normal GM (*n* = 35). Using DXA, BMD, TBS_BMI_, TBS_TT_, and VF screening were performed.

**Results:**

In all subjects, TBS_TT_ was higher (mean 9.5%) than TBS_BMI_. Abnormal GM subjects had lower TBS_BMI_ (1.166 ± 0.15) than normal GM subjects (1.232 ± 0.12; *p* < 0.05). No between-group difference in TBS_TT_ or BMD was observed. In a multiple regression model, the best predictor of TBS_TT_ was HbA1c (*p* = 0.002). None of the DXA measures or GM parameters was a significant predictor of VF (*n* = 7).

**Conclusion:**

The abnormal GM acromegaly subjects had lower TBS_BMI_ than those with normal GM. TBS_TT_ was higher than TBS_BMI_, and no between-group difference based on GM status was observed. TBS_TT_ was significantly associated with GM parameters, notably HbA1c. The relationship of TBS_TT_ with GM parameters may imply an effect of GM on trabecular bone microstructure in patients with acromegaly; a further study is indicated.

## Introduction

In those with acromegaly, chronic GH hypersecretion leads to increased fasting plasma glucose (FPG), impaired insulin tolerance, and diabetes similar to type 2 diabetes (T2DM). T2DM adversely affects the skeleton with low bone turnover, accumulation of advanced glycation end products, and micro- and macro-architecture alterations that lead to impaired strength (1). Despite this, many studies find the bone mineral density (BMD) in T2DM patients to be ~5%–10% higher than those of age-matched non-diabetic populations (2). This BMD elevation is more pronounced in those with high BMI and, perhaps surprisingly, high HbA_1_c levels. This enhancement in BMD is predominantly a feature of the weight-bearing skeleton but not of non-weight-bearing bone such as the forearm. However, some caveats exist regarding the greater spine BMD in patients with T2DM, as diffuse idiopathic skeletal hyperostosis is common (3) and elevates DXA-measured BMD. By contrast, the lumbar spine trabecular bone score (TBS) is somewhat lower in patients with T2DM (4–6). A meta-analysis of 2,018 patients with T2DM found a lower TBS in diabetic than non-diabetic individuals, suggesting that TBS can be a useful measurement for fracture risk assessment in diabetic patients (7). Previously, it was thought that acromegaly patients have low BMD (8). More recently, it has been revealed that GH hypersecretion is associated with normal or even higher BMD (9), which is partly explained by larger bone size and differing effects on cortical and trabecular bone (10–13). However, several quantitative computed tomography studies in acromegaly patients found poor trabecular bone parameters, such as higher trabecular separation, lower trabecular number, and lower bone volume to tissue volume, in comparison with healthy controls (12). It is plausible that adverse trabecular bone status among acromegalic patients is related to glucose status, as a negative association of TBS with GM was indicated in the sub-analysis of a recent case–control study (14). However, there is a lack of studies that comprehensively evaluate the effect of GM status on bone parameters as assessed by DXA in acromegaly patients.

Acromegaly subjects have greater soft tissue due to the effect of GH on soft tissues such as muscle, cartilage, and visceral fat (15, 16). Thus, the potential effects of soft tissue thickness on bone measurements provided by DXA should be considered. Importantly, TBS may be lowered by greater soft tissue thickness and, as such, is currently adjusted by BMI, denoted as TBS_BMI_ (17). Moreover, greater soft tissue thickness is associated with higher fracture risk (18). Forthcoming TBS software (TBS_TT_) directly adjusts TBS for densitometer-measured tissue thickness. While not currently available for clinical use, TBS_TT_ is increasingly being reported in research studies (20–24). However, TBS_TT_ results have not been reported in those with acromegaly.

Thus, the aim of this study was to comprehensively analyze DXA-measured BMD, TBS_BMI_, and TBS_TT_ and evaluate their association with GM in patients with acromegaly.

## Patients and methods

This single-center cross-sectional study of acromegaly patients was conducted at the National Institute of Endocrinology and Diabetology, Lubochna, Slovakia. Acromegaly patients evaluated from June 2016 to February 2020 were included. The regional medical ethics committee approved this study; signed informed consent was obtained prior to the conduct of any study procedure.

The inclusion criteria were as follows:

- Diagnosis of acromegaly, i.e., GH hypersecretion defined as failure to suppress GH concentration to below 1 ug/L following documented hyperglycemia during oral glucose load and increased IGF-1 (25).
- All patients regardless of age, sex, disease activity, or acromegaly-associated treatments were included in this study.

Hypopituitarism was defined as having at least one pituitary axis insufficiency and was diagnosed and treated according to the Endocrine Society (ES) guidelines (26). Central hypothyroidism was defined as free thyroxine (fT4) level below the reference range in conjunction with low or normal thyroid-stimulating hormone (TSH). Central adrenal insufficiency was defined as 8–9 a.m. serum cortisol values below 80 nmol/L or failure to increase the cortisol levels to more than 500 nmol/L at 60 min during corticotropin (ACTH) stimulation test. Male hypogonadism was defined as serum testosterone (TST) concentration and/or sperm count below the lower limit of normal with serum luteinizing hormone (LH) and follicle-stimulating hormone (FSH) concentrations not being elevated. In female patients, central hypogonadism was diagnosed by the presence of oligomenorrhea or amenorrhea, no history of uterine instrumentation or pregnancy, and normal or decreased FSH and/or LH in the absence of hyperprolactinemia (27). Diabetes insipidus was diagnosed by the presence of polyuria plus high serum osmolarity (more than 295 mOsm/L) and urine osmolality/plasma osmolality ratio ≥2.

### Exclusion criteria

- Active pharmacologic osteoporosis treatment: Supplementation with vitamin D and calcium in the dose recommended by ESCEO/IOF was allowed (28).
- Patients with BMI outside of the TBS_BMI_ recommended range (15–37 kg/m^2^).

The following parameters were examined in all of the patients:

1. Anthropometric parameters: weight (kg), height (cm), and body mass index (BMI; kg/m^2^).
2. Disease activity: According to the ES guidelines (25), active disease was defined by an IGF-1 level above the upper limit of normal for age regardless of treatment modality and a GH level >1 g/L with failure to suppress GH to <1 g/L after oral glucose load. Otherwise, the disease was considered controlled. The IGF-1 levels were measured using IMMULITE^®^ 2500 assay, a solid phase, enzyme-labeled chemiluminescent immunometric method (ECLIA) with inter-assay variability CV of 2.4%–4.7%. The GH levels were measured with an immunoassay ECLIA having a sensitivity of 0.05 μg/L.
3. Hormonal status: Pituitary and their target hormone (TST, LH, FSH, PRL, morning serum cortisol, ACTH, TSH, and fT4) levels were measured in a routine clinical manner. All blood samples were obtained between 7:00 and 8:00 following at least an 8-h fast.
4. Bone measurements: Each subject had BMD and TBS measurement performed ±7 days from blood sampling. BMD measurements at the L1-4 spine, femoral neck (FN), and total hip (TH) were performed using a Hologic Horizon device with APEX software version 13.3:7. L1-4 TBS was performed initially using TBS iNsight^®^ software (Medimaps SASU, Pessac, France), version 3.0.2.0. Subsequently, blinded TBS_TT_ analysis was performed using TBS iNsight software 4.0. centrally in Geneva, Switzerland. Briefly, the TBS_TT_ algorithm was derived through experimental work, where scans of dry *ex vivo* human vertebrae with different thicknesses of soft tissue equivalent material were acquired. Then, the relationship between TBS and soft tissue thickness was estimated and a specific model was defined and further applied to *in vivo* data (19). Vertebra were excluded from BMD and TBS analysis when there was more than one SD difference in BMD T-score from the immediately adjacent vertebral body and if vertebral fracture or cementoplasty were present.
5. GM parameters: Fasting blood glucose, HBA1c (DCCT; Diabetes Control and Complication Trial), C-peptide, and insulin resistance index (IRI) were assessed in a routine clinical manner.
6. VF assessment was performed on lateral spine images obtained using a Hologic Horizon^®^ A densitometer (Hologic Inc., Marlborough, MA, USA) in supine position. VF was defined using the Genant VSQ approach (29) by Professor Harry Genant with vertebral height reduction of 20%–25% defining grade I, 26%–40% grade II, and ≥40% grade III. A subject was considered “fractured” if a fracture was observed regardless of unreadable levels elsewhere. VFA obtained at baseline defined prevalent fractures; new VFs at the end of the follow-up were considered incident VFs.

The subjects included in this analysis were divided into two groups based on the American Diabetes Association (ADA) 2016 guidelines (30):

1. patients with abnormal GM.
2. patients with normal GM.

Abnormal GM was defined as overt DM or prediabetes where we included those with borderline fasting blood glucose (5.6–6.9 mmol/L or 140–200 mg/dL) or impaired glucose tolerance (7.8–11.1 mmol/L at hour 2 of oGTT) or increased HBA1c (5.7%–6.4%).

### Statistics

Statistical analysis was performed using the software JASP (University of Amsterdam, Netherlands, v.0.18.3). The obtained data were expressed as mean ± standard deviations (SD). Kolmogorov normality test was used to test the normality of the data. Comparisons between normal GM and abnormal GM cohorts were derived from Student’s *t*-test for continuous variables with normal distribution, Wilcoxon–Mann–Whitney test for continuous variables with not normal distribution, and chi-square test for categorical variables. Correlations between the investigated continuous parameters were calculated using Pearson correlation coefficient. Linear regression analysis and logistic regression were used to calculate possible relationships between significant parameters and TBS TT or vertebral fractures, respectively. The threshold for statistical significance (*p*-value) was set at 5%.

## Results

### Study group’s characteristics

A total of 70 patients—24 male (34%) and 46 female (66%)—were included in this analysis. Their mean ± SD age was 55.1 ± 11.6 years. A total of 35 (50%; 14 M/21 F) had abnormal GM, and 35 (50%; 10 M/25 F) had normal GM. At the time of diagnosis, acromegaly was treated surgically in most patients (71%), followed by medical treatment with somatostatin receptor ligands (SRLs) in 55 subjects (78%). SRL treatment was used in 34 (97%) of subjects in the abnormal GM group and 21 (60%) in the normal GM group. Out of 35 patients with abnormal GM, eight patients (22%) were treated with antidiabetic medication: five patients had oral antidiabetic therapy (metformin average dose = 1,500 mg/day), two patients had insulin, and one patient was on combined treatment with insulin + oral antidiabetic (metformin 2,000 mg/day). In the group with abnormal GM, 15 (42.8%) subjects had active disease, 23 (65.7%) had undergone pituitary surgery, eight (22.8%) were treated with SRLs pre-operatively, and 26 (74.2%) were treated with SRLs post-operatively. Among the normal GM group, 11 (31.4%) had active disease, 27 (77%) had pituitary surgery, 10 (28.5%) were treated with SRLs pre-operatively, and 14 (40%) were treated with SRLs post-operatively. A greater number of subjects within the abnormal GM group (*N* = 26) were treated with SRLs post-operatively than in those with normal GM (*N* = 14) (*p* < 0.001). The subjects within the abnormal GM group had a longer duration of SRL treatment pre- and post-operatively (both *p* < 0.05) (see also **Table 1**). The subjects with abnormal GM had greater weight (92.8 ± 18.9 vs. 82.7 ± 19.1 kg; *p* < 0.05) and BMI (33.1 ± 12.6 vs. 28.2 ± 5.5 kg/m^2^; *p* < 0.05) than subjects with normal GM. A longer duration of treatment with SRLs among subjects with abnormal GM was observed (see **Table 1**). The subjects with abnormal GM had significantly higher values of HGBA1c (6.25% ± 1.13% vs. 5.5% ± 0.41%) and fasting blood glucose (6.46 ± 1.36 vs. 5.1 ± 0.26 mmol/L) than the patients with normal GM (both *p* < 0.05, see **Table 1**). There was no difference in C-peptide IRI or IGF1 levels between groups.

**Table 1:** Baseline characteristics of the study groups—comparison of subjects with impaired vs. normal GM.

	All (*n* = 70)	abnormal GM (*n* = 35)	normal GM (*n* = 35)
**Age (y**ea**rs)**	55.1 ± 11.1	54 ± 12.4	57.2 ± 9.3
**Weight (kg)**	87.9 ± 18.9	92.8 ± 18.9	82.7 ± 19.1*
**Height (cm)**	170.7 ± 12.8	170.0 ± 16.6	172.3 ± 9.7
**BMI (kg/m^2^)**	30.7 ± 9.9	33.1 ± 12.6	28.2 ± 5.5*
**Male/female**	24/46	14/21	10/25
**IGF1 (ng/mL)**	259.6 ± 202	243 ± 122	233 ± 231
**Active/controlled disease, *n* (%)**	26(37.1)/44 (62.9)	15 (42.8)/20 (57.2)	11 (31.4)/24 (68.6)
**Pitu**i**tary surgery, *n* (%)**	50 (72.5)	23 (65.7)	27 (77)
**SRLs pre-operative treatment**, ***n* (%)**	18 (26.5)	8 (22.8)	10 (28.5)
**SRLs post-operative, *n* (%)**	40 (65.6)	26 (74.2)	14 (40) *
**SRLs pre-operative treatment duration (months)**	37.9 ± 43.8	66.2 ± 49	29.4 ± 47*
**SRLs post-operative treatment (months)**	63.1 ± 52.532	74.8 ± 46.8	44.8 ± 57.1*
**Dopamine agonist treatment, *n* (%)**	24 (34.2)	16 (45.7)	8 (22.8)
**Pegvisomant treatment, *n* (%)**	15 (24.2)	10 (28.5)	5 (14.2)
**Radiotherapy, *n* (%)**	33 (0.47)	21 (60)	12 (34.2)
**Central hypogonadism, *n* (%)**	27 (38.6)	15 (42.8)	12 (34.2)
**Central hypothyroidism, *n* (%)**	22 (33.3)	10 (28.5)	12 (34.2)
**ACTH deficiency, *n* (%)**	15 (22.7)	6 (18.1)	9 (25.7)
**GH deficiency, *n* (%)**	3 (4.3)	0	3 (8.5)
**Fasting plasma glucose (mmol/**L)	5.8 ± 1.27	6.46 ± 1.36	5.1 ± 0.26*
**C-peptide**	0.605 ± 0.4	0.653 ± 0.31	0.526 ± 0.56
**HbA1c (%)**	5.92 ± 0.93	6.25 ± 1.13	5.5 ± 0.41*
**HbA1c ≥ 6.5%, *n* (%)**	9 (12.8)	9 (25.7)	0*
**IRI**	13.09 ± 20	15.1 ± 11.1	11.9 ± 20.8
**25(OH)D (nmol/L)**	68.9 ± 27.1	67.9 ± 24	70.6 ± 30
**Calcium (mmol/L)**	2.35 ± 0.03	2.34 ± 0.03	2.37 ± 0.03
**CTX (pg/mL)**	427.3 ± 219	414 ± 202	459 ± 243
**P1NP (ug/L)**	57.3 ± 29.5	60 ± 33	55 ± 28
**L-**s**pine BMD (g/cm^2^)**	0.974 ± 0.156	1.011 ± 0.147	1.029 ± 0.182
**TH BMD (g/cm^2^)**	1.004 ± 0.159	0.984 ± 0.148	0.964 ± 0.155
**TBS_BMI_**	1.198 ± 0.14	1.166 ± 0.15	1.232 ± 0.12*
**TBS_TT_**	1.324 ± 0.086	1.324 ± 0.087	1.316 ± 0.085
**Subjects with vertebral fractures, *n* (%)**	7 (10)	5 (14.2)	2 (5.7)
**Subjects with multiple vertebral fractures, *n* (%)**	5 (7.1)	3 (8.5)	2 (5.7)

Data are mean (SD). The asterisk represents *P* ≤ 0.05 for the comparisons between normal GM and abnormal GM cohort, which were derived from Student’s *t*-test (continuous variables with normal distribution), Wilcoxon–Mann–Whitney test (continuous variables with not normal distribution), and chi-square test (categorical variables).

GM, glucose metabolism; *n*, number; IGF-1, insulin-like growth factor 1; BMI, body mass index; SRL, first-generation somatostatin receptor ligand; ACTH, adrenocorticotrophic hormone; GH, growth hormone; HbA1c, glycosylated hemoglobin; IRI, insulin resistance index; CTx, C-carboxyterminal collagen crosslinks; P1NP, procollagen 1 N-terminal peptide; L-spine, lumbar spine; BMD, bone mineral density; TH, total hip; TBS, trabecular bone score.

### Bone measurements

**Figure 1:**
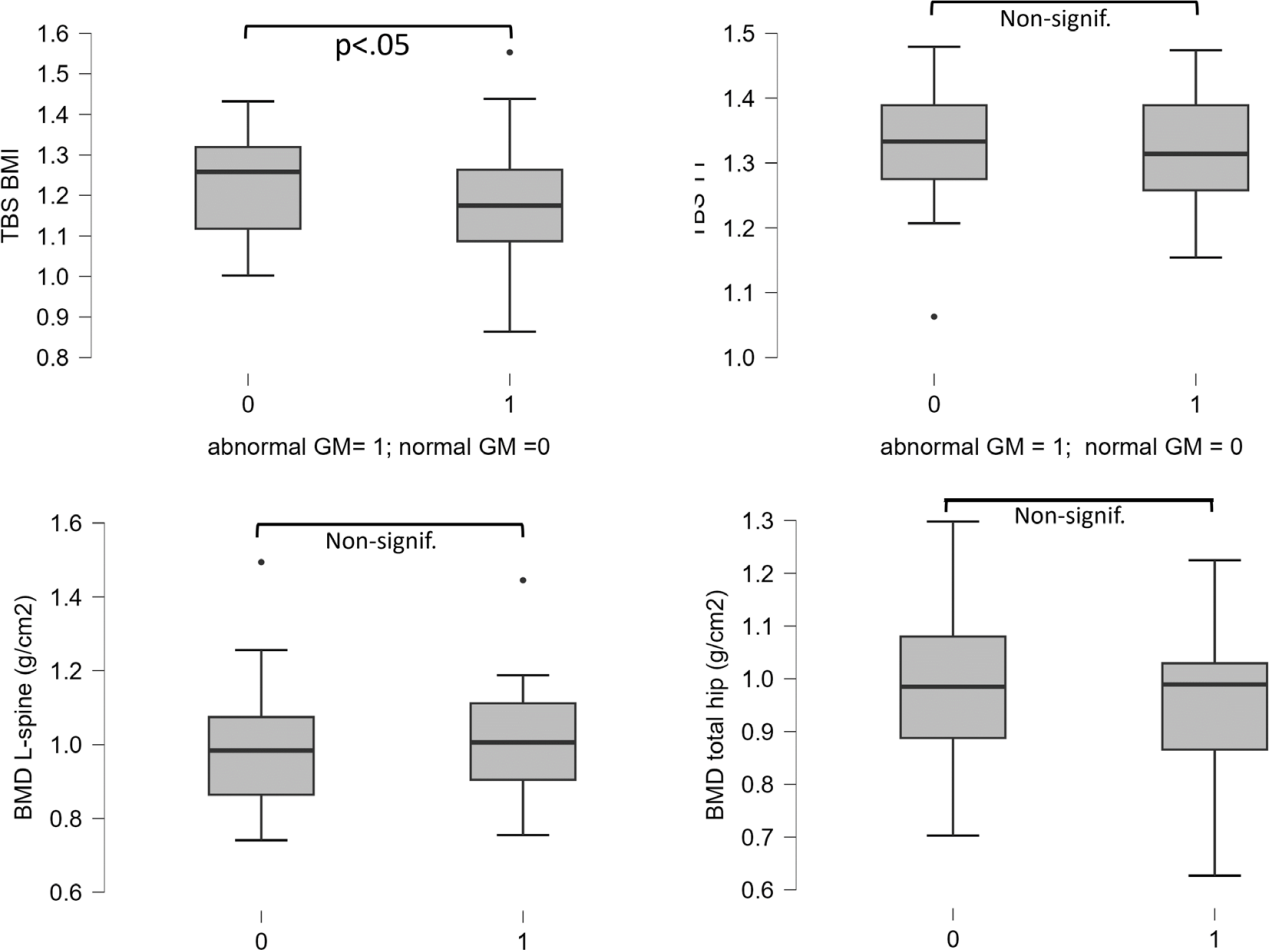
Differences of TBS_BMI_, TBS_TT_, L-spine BMD, and total hip BMD between subgroups with abnormal and normal glucose metabolism. A significant difference of TBS_BMI_ was observed.

In all subjects, regardless of GM status, TBS_TT_ (mean 1.324 ± 0.09) was higher by ~10% than TBS_BMI_ (mean 1.198 ± 0.14) (*p* < 0.001). Patients with abnormal GM had a significantly lower mean TBS_BMI_ than those with normal GM (1.166 ± 0.15 vs. 1.232 ± 0.12; *p* < 0.05). TBSTT did not differ by GM status; TBS_TT_ = 1.324 ± 0.087 in the abnormal GM group vs. 1.316 ± 0.085 in the normal GM group (**Figure 1**). No difference in BMD or in bone turnover markers between patients with and without GM was observed.

IGF-1, FPG, HBA1c, C-peptide, duration of SRL pre- and post-operatively, TBS_TT_, and TBS_BMI_ were correlated in a simple regression model (see **Table 2**). A significant correlation of IGF-1 with C-peptide (*R* = 0.293; *p* = 0.02), but not with other parameters, was observed. HBA1c (*R* = 0.802; *p* < 0.001) and FPG (*R* = 0.572; *p* = 0.01) were significantly associated with pre-operative SRL treatment duration. FPG (*R* = - 0.402; *p* < 0.001), HbA1c (*R* = -0.474; *p* < 0.001), and C-peptide (*R* = -0.341; *p* = 0.007) were negatively associated with TBS_BMI._ A weaker correlation of FPG (-0.244; *p* = 0.05) and HBA1c (*R* = -0.331; *p* < 0.007) with TBS_TT_ was observed (see **Table 2**).

**Table 2:** Correlation matrix for simple regression models between significant parameters—FPG, HbA1c, C-peptide, IGF-1, duration of SRL pre- and post-operative treatments, TBS_BMI_, and TBS_TT_.

Pearson's Correlations								
Variable		Fasting plasma glucose (mmol/L)	HBA1c (%)	C-peptide (nmol/L)	IGF-1 (ng/mL)	Duration of SRLs pre-operatively (months)	TBS TT	TBS BMI
Fasting plasma glucose (mmol/L)	Pearson's *r*	—						
	*p*-value	—						
HBA1c (%)	Pearson's *r*	**0.712**	—					
	*p*-value	**<0.001**	—					
C-peptide (nmol/L)	Pearson's *r*	**0.271**	0.116	—				
	*p*-value	**0.035**	0.384	—				
IGF-1 (ng/mL)	Pearson's *r*	0.154	-0.102	**0.293**	—			
	*p*-value	0.213	0.414	**0.020**	—			
Duration of SRLs pre-operatively (months)	Pearson's *r*	**0.572**	**0.802**	-0.060	-0.203	—		
	*p*-value	**0.010**	**<0.001**	0.814	0.390	—		
Duration of SRLs post-operatively (months)	Pearson's *r*	-0.083	0.043	-0.092	0.775	—		
	*p*-value	0.612	0.795	0.584	0.225	—		
TBS TT	Pearson's *r*	**-0.244**	**-0.331**	-0.217	-0.037	-0.143	—	
	*p*-value	**0.047**	**0.007**	0.087	0.758	0.547	—	
TBS BMI	Pearson's *r*	**-0.402**	**-0.474**	**-0.341**	-0.008	-0.271	**0.887**	—
	*p*-value	**<0.001**	**<0.001**	**0.007**	0.948	0.261	**<0.001**	—

Significant associations are highlighted in bold.

According to a linear regression model including all parameters (FPG, HbA1c, C-peptide, preoperative duration of SRL treatment, and weight), the best predictors for TBS_TT_ was HbA1c (*p* = 0.002) and duration of SRL treatment preoperatively (*p* = 0.03) (see **Table 3**).

**Table 3:** Multiple regression model. According to a linear regression model with all of the parameters included (FPG, HbA1c, C-peptide, preoperative duration of SRL treatment, and weight), the best predictor for TBS_TT_ was HbA1c.

Model	Unstandardized	Standard error	*p*
Fasting plasma glucose (mmol/L)	0.022	0.016	0.197
HBA1c (%)	-0.193	0.048	0.002
C-peptide (nmol/L)	0.133	0.085	0.143
SRLs, preoperative duration (months)	0.002	0.00083	0.029
Weight (kg)	-0.001	0.001	0.324

### Vertebral fractures

**Figure 2:**
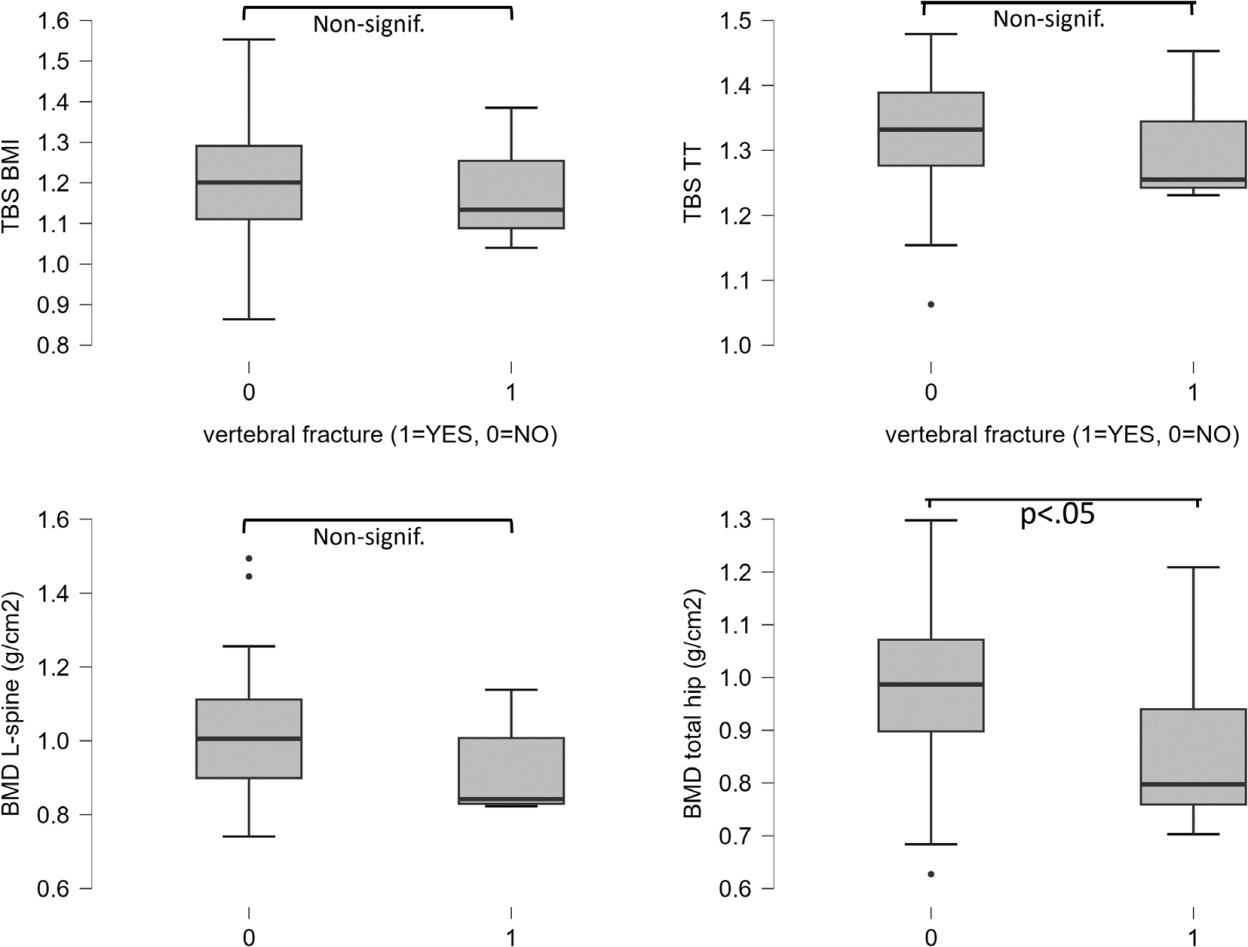
Differences of TBS_BMI_, TBS_TT_, L-spine BMD, and total hip BMD between subgroups with vertebral fracture (VF) and without VF. A significant difference of total hip BMD was observed.

There were seven subjects with asymptomatic vertebral fracture (VF) in the entire cohort, of which five were in the abnormal GM group. There were five subjects with multiple VFs. No difference in the prevalence of VFs or multiple VFs between groups with abnormal and normal GM was observed (see **Table 1**). Total hip BMD was significantly lower in those with VF (0.874 ± 0.19 g/cm^2^) than in those without VF (0.985 ± 0.15 g/cm^2^; *p* < 0.05). No difference in TBS_TT_, TBS_BMI_, or L-spine BMD between the VF and non-VF groups was observed (see **Figure 2**). Among all GM or lumbar spine DXA parameters, none was a significant predictor of VF as assessed by logistic regression (see **Table 4**).

**Table 4:** Logistic regression model. From all glucose metabolism parameters, TBS_BMI_, TBS_TT_, and BMD, none was a significant estimate for vertebral fracture.

	Estimate	Standard error	*p*
Fasting plasma glucose (mmol/L)	0.725	0.631	0.250
HBA1c (%)	-0.561	1.004	0.576
C-peptide (nmol/L)	-0.616	1.884	0.744
IGF-1 (ng/mL)	-0.001	0.003	0.724
TBS BMI	1.905	8.286	0.818
TBS TT	-1.954	16.086	0.903
BMD L-spine (g/cm^2^)	-0.624	4.930	0.899
BMD total hip (g/cm^2^)	-3.383	4.710	0.473

## Discussion

In this study, acromegaly patients with abnormal GM have lower TBS_BMI_ than those with normal GM. Correction of TBS for tissue thickness (TBS_TT_) increased the TBS values by ~10% compared to TBS_BMI_, and no difference in TBS_TT_ according to GM status was observed. TBS_TT_ was still negatively associated with fasting plasma glucose and HBA1c. Several significant associations between GM, IGF-1, duration of SRL treatment, TBS_BMI_, and TBS_TT_ were observed. From all of these parameters, multiple regression analysis found HbA1c to be the most significant predictor of TBS_TT_. In addition, there was a relationship between GM and duration of SRL treatment, which is possibly one of the risk factors for impaired glucose metabolism. The relationship of TBS_TT_ with the parameters of GM status suggests that trabecular bone microstructure in acromegaly might be affected by abnormal GM, not simply GH hypersecretion itself. VF prevalence did not differ between groups according to GM status, which was likely due to the small number of fractures. Finally, this study demonstrates that TBS correction for soft tissue thickness significantly affects the result; this should be considered in conditions with higher amounts of soft tissue, such as acromegaly, diabetes, obesity, etc.

Diabetes mellitus occurs in about 20%–40% of patients with acromegaly (31); the clinical picture resembles type 2 diabetes mellitus (32). In this study, we confirmed a high prevalence of GM impairment among subjects with acromegaly. Patients with acromegaly and abnormal GM have lower TBS_BMI_, but not TBS_TT_, than those with normal GM. Despite the differences in BMD, the risk of fractures in patients with diabetes mellitus type 1 and type 2 is increased (33). Normal or slightly increased BMD in patients with type 2 diabetes mellitus does not “protect” them from osteoporotic fractures, suggesting that BMD alone underestimates fracture risk in such individuals. As with other forms of secondary osteoporosis (34–37), the bone quality in acromegaly does not correspond to BMD. Although we confirmed a difference in TBS_BMI_, but not BMD, in the observed groups, the prevalence of VF in our cohort did not differ significantly based on the occurrence of GM disorder. In a simple regression model, we confirmed a positive correlation between IGF-1 and C-peptide, which indicates a link between GM and GH hypersecretion. At the same time, a significant negative association between TBS_TT_ and GM parameters was confirmed. In a study of 105 postmenopausal women with type 2 diabetes mellitus, the analysis showed that HBA1c below 7.0% had no effect on BMD in patients with type 2 diabetes mellitus, but at the same time, patients with HBA1c <7.0% had better TBS_BMI_ than patients with HBA1c above 7.0%. In the group of 14 patients with a low traumatic clinical fracture in different locations, higher HBA1c and lower TBS_BMI_ compared to patients without clinical fracture were observed (38). However, there was no difference between TBS_TT_ in this study according to GM impairment. Thus, other factors that could influence both glucose metabolism and soft tissue also come into consideration. Because we saw a strong correlation of SRL treatment duration pre-operatively, we must admit that SRLs have a significant impact on GM. In addition, in the group with abnormal GM, a greater number of subjects were treated with SRLs post-surgery. It is well known that SRLs act on pancreatic alpha‐ and beta‐cells, impairing both insulin and glucagon secretion. Despite many studies demonstrating that SRL administration leads to impairment of glucose homeostasis, there are some studies proving that the effect on glucose metabolism is not statistically significant (39–41). Based on our analysis, SRLs likely play a certain role in worsening glucose metabolism and thus indirectly lead to a decline in TBS_BMI_ or tissue thickness. However, this should be analyzed by longitudinal studies aimed specifically to assess the effect of SRLs on bone microarchitecture.

Overall, the relationship between GM and its effect on bone in patients with acromegaly is addressed only by a minimum of studies (42). To our knowledge, this study is the only available publication that discusses the influence of GM parameters on bone parameters among subjects with acromegaly.

This study has several strengths, such as a relatively large sample size considering that acromegaly is a rare diagnosis. Multiple DXA parameters were evaluated, and vertebral fractures were identified by Dr. Harry Genant^†^. All DXA parameters evaluated are potentially available in routine clinical practice. Additionally, TBS correction for soft tissue thickness (TBS_TT_) was used. Undoubtedly, TBS_TT_ brings new light to the issue of fracture risk assessment and must be considered in subjects with greater abdominal soft tissue. Study limitations include the absence of a control group; a comparison of acromegaly diabetic subjects with the general diabetic population could also improve this study. Finally, we were unable to clarify the relationship between the onset of GM impairment and bone status.

In conclusion, this study showed that acromegaly subjects with abnormal GM have lower TBS_BMI_ compared to those with normal GM, and after correction of TBS_BMI_ for soft tissue thickness, this difference was lost and, importantly, TBS_TT_ increased significantly. However, TBSTT remained significantly associated with GM, mostly HbA1c. This study suggests, for the first time, that trabecular bone microstructure, as indirectly assessed by TBS_TT_, might be affected by abnormal GM, not GH hypersecretion itself. In addition, SRL treatment likely plays a role. However, further studies with a greater number of subjects and with comparison between healthy controls and subjects with diabetes among the general population are needed to better understand this issue.

## Data Availability

The datasets presented in this article are not readily available because it contains personal data. Requests to access the datasets should be directed to kuzma@ru.unb.sk.
